# Regulation of the Biosynthesis of Phenolic Compounds in *Vaccinium* Berries

**DOI:** 10.1021/acs.jafc.6c08282

**Published:** 2026-09-25

**Authors:** Laura Jaakola

**Affiliations:** † Department of Arctic and Marine Biology, UiT the Arctic University of Norway, Tromsø NO-9037, Norway; ‡ Division of Food Production and Society, Norwegian Institute of Bioeconomy Research (NIBIO), Ås NO-1431, Norway

**Keywords:** anthocyanins, berries, flavonoids, phenolic compounds, *Vaccinium*

## Abstract

*Vaccinium* berries are among the food ingredients with the highest levels and most diverse compositions of phenolic compounds, including anthocyanins, proanthocyanidins, and flavonols along with hydroxycinnamic acids. The biosynthesis of these compounds through the phenylpropanoid pathway is regulated via coordinated transcriptional control of the structural enzymes in the pathway by the MBW complex that involves interactions with DNA-binding R2R3MYB transcription factors, MYC-like basic helix loop helix (bHLH), and WD40-repeat proteins. Some upstream regulators of the pathway were also identified. Both the developmental process and environmental factors influence the metabolism of phenolic compounds during berry ripening. Recent studies have deepened our understanding of the complex regulatory networks controlling the berry ripening-related biosynthesis of phenolic compounds. This knowledge enables the development of new tools for the selection of cultivars with desirable traits, such as berries with enhanced health benefits or cultivars with greater stress resistance.

## Graphical abstract

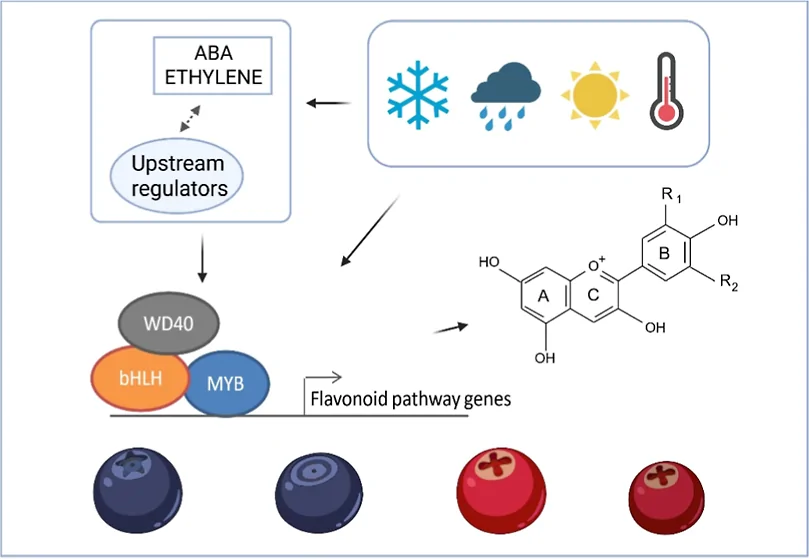

## 1 Introduction

*Vaccinium* species encompass many economically important cultivated and wild berries, such as blueberries (*Vaccinium corymbosum*, *V. virgatum*, *V. angustifolium*), cranberries (*V. macrocarpon*), bilberries (*V. myrtillus*), and lingonberries (*V. vitis-idaea*). These species grow in acidic soils and mostly in cooler northern hemisphere climates.^1^ Multiple studies have shown evidence on the health beneficial effects of *Vaccinium* berries, for example, in reducing risk of metabolic syndrome and various degenerative and microbial diseases.^2,3^ Health benefits are mostly attributed to high level of phenolic compounds present in these berries, even if they are also good sources of other health beneficial compounds, such as vitamins, carotenoids, and fibers. Recently, a substantial amount of new genetic and functional data has been produced on the regulation of phenolic compounds in *Vaccinium* species. This review considers the update on the biosynthesis of the phenolic compounds present in wild- and cultivated *Vaccinium* berries. The special focus is on the recent progress in understanding the regulation of the biosynthesis of anthocyanins and other key compounds of the flavonoid pathway during berry development and ripening and as response to environmental cues.

## 2 Vaccinium Phenolics

In *Vaccinium* species, anthocyanins, proanthocyanidins, and flavonols are flavonoid classes contributing to the main phenolics in addition to hydroxycinnamic acids.^2,4,5^ Flavonoids in general are the most common phenolic compounds in fruits, all of which are built upon the same C15 (C6–C3–C6) basic skeleton in which the three-carbon bridge is cyclized with oxygen. Flavonoid classes differ in the oxidation level of the central pyran ring except chalcones, which have an open central pyran (Figure 1).

**Figure 1:**
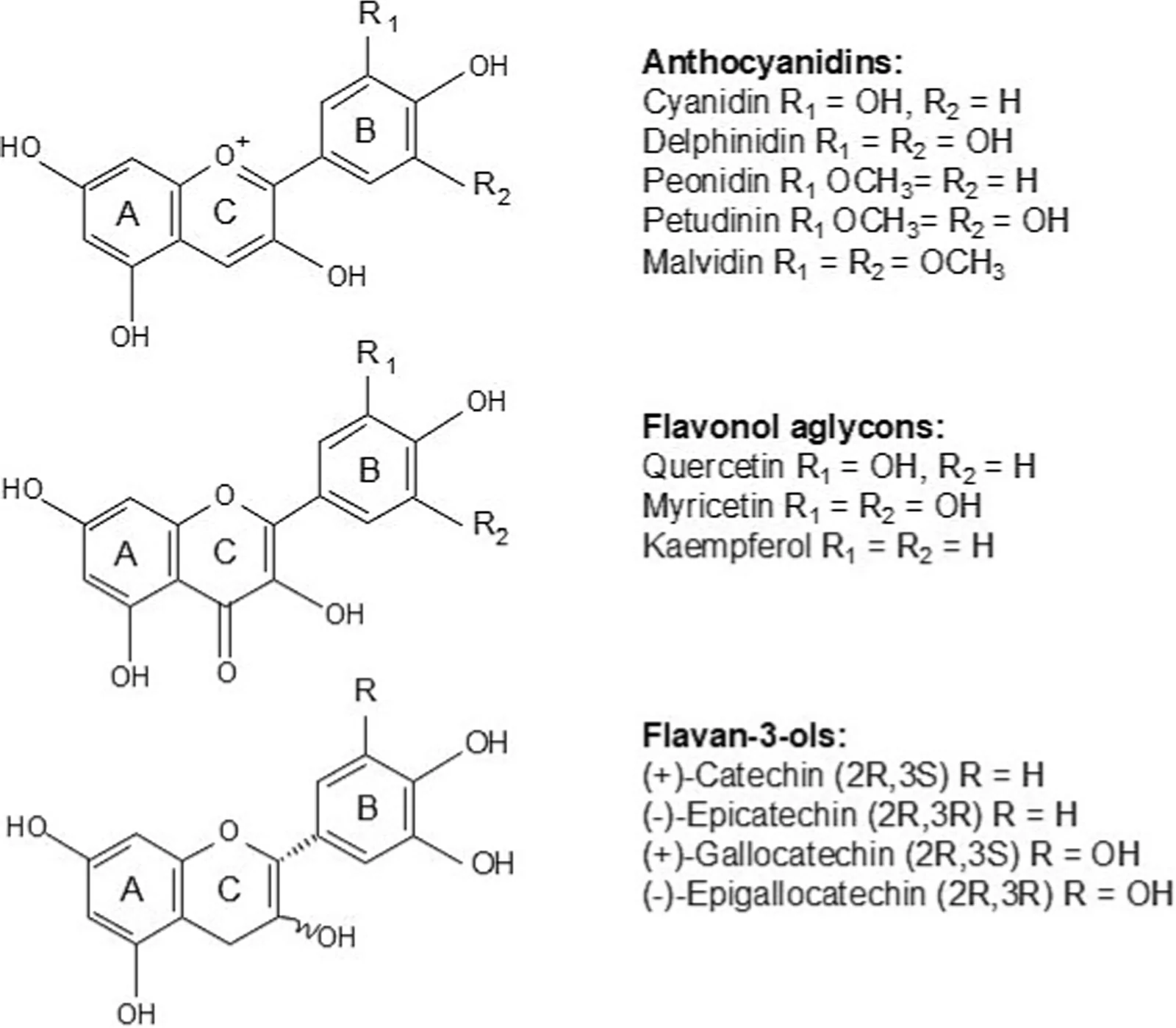
Chemical structures of the most common flavonoids in*Vaccinium* berries.

*Vaccinium* species contain high levels of hydroxycinnamic acids (HCAs), which can be found in all organs and represent the main phenolics in the leaves. HCAs are found in plant tissues in combined forms, often linked through an ester bond between their carboxylic group and an alcohol group of an organic compound or through a bond with one of their phenolic groups. HCAs can also be associated with flavonoids, lignin, suberin, and cutin. In most *Vaccinium* berries, caffeic, ferulic, and *p*-coumaric acids have all been identified.^2,5,6^

Flavonols (3-hydroxyflavones) are distinguished by a double bond between the C-2 and C-3 positions along with a hydroxyl group at the C-3 position.^7^ These compounds are primarily found as glycosides, with the sugar moiety typically attached to the hydroxyl group at the 3-position. The possible sugar residues include d-glucose, d-galactose, l-rhamnose, l-arabinose, d-xylose, and d-glucuronic acid. Quercetin glycosides are the most common flavonols present in all *Vaccinium* berries and also in early developmental stages. Myricetin glycosides are found more abundantly in ripe, blue-colored berries, while kaempferol glycosides are present in smaller amounts (Figure 1).^4,8^ In leaves, quercetin and kaempferol glycosides are the dominant flavonols.^5^

Anthocyanins are the primary pigments in *Vaccinium* species, responsible for the bluish and reddish hues of the berries. The accumulation of anthocyanins is rapid during ripening stage attracting seed dispersals and enhance the quality and value of ripe berries by influencing their appearance and nutritional properties. Structurally, anthocyanins consist of an anthocyanidin aglycone attached to one or more sugar molecules. To date, over 700 distinct anthocyanins have been identified, with the most significant being glucosides of six common aglycones: cyanidins, delphinidins, pelargonidins, petunidins, peonidins, and malvidins.^9^ Among these, cyanidins, delphinidins, petunidins, peonidins, and malvidins are typically found in blue-colored *Vaccinium* berries, while red-colored cranberries and lingonberries primarily contain cyanidin-derived anthocyanins, specifically cyanidin and peonidin glucosides (Figure 1).^2,10^ The levels of anthocyanins in *Vaccinium* berries vary largely between species but also within species affected by the ripening stage, environmental factors, and genetic variation. Bilberries generally contain 4–5 fold higher levels of anthocyanins compared to blueberries, due to the high anthocyanin content in flesh, whereas in most blueberry species anthocyanins are only found in skin.^5^ Moreover, further decorations of anthocyanins, such as acylation, have also been reported especially in blue-colored *Vaccinium* berries, such as blueberries, bilberries, and bog bilberries.^11–13^ Numerous studies associate anthocyanins with protective effects against human diseases, including cardiovascular conditions, age-related degenerative diseases, obesity, metabolic syndrome, and cancer.^2,3^ It has also been shown that a key mechanism behind these health benefits is the significant influence of an anthocyanin-rich diet on gut microflora, which further processes these compounds into functional end products.^14–16^

Proanthocyanidins (PAs), also termed condensed tannins, are the most structurally complex members of the flavonoid family, ranging from dimers to high-molecular-weight polymers. These polymers are composed of flavan-3-ols as their structural units, which also exist as monomers in the plant tissues. In *Vaccinium* species, the most commonly identified flavan-3-ols include (+)-catechin (2,3-trans-), (−)-epicatechin (2,3-cis-), (+)-gallocatechin, and (+)-epigallocatechin (Figure 1).^17^ The structural diversity of proanthocyanidins arises from variations in the stereochemistry and hydroxylation patterns of the flavan-3-ol starter and extension units.^18^ The most prevalent flavanol–flavanol linkages are carbon–carbon bonds between the 4-position of the upper unit and the 6- or 8-position of the lower unit, known as B-type linkages. Less common A-type linkages involve a C4 → C8 bond along with an ether bond O7 → C2. Although A-type linkages are rare in fruits, both A- and B-type linkages have been identified in *Vaccinium* species. During berry development, the level of PAs is highest during the earlier developmental stages and decreases during the ripening.^17^ As astringent compounds, PAs provide protection for the developing seeds inside berry against early feeding of the berries. PAs are the most abundant flavonoids in cranberry fruits and are associated with their potential health benefits, such as preventing urinary tract infections and treating oral infections.^19^

## 3 Biosynthesis of Phenolic Compounds

The shikimate pathway serves as the starting point for the biosynthesis of the phenolic compounds. The general phenylpropanoid pathway begins with phenylalanine, which is converted into cinnamate (*trans*-cinnamic acid) by the enzyme phenylalanine ammonia lyase (PAL) (Figure 2). Cinnamic acid acts as a key intermediate in both the phenylpropanoid and shikimate pathways and serves as a precursor for its hydroxyl derivatives, including *p*-coumaric, caffeic, ferulic, sinapic, and chlorogenic acids. The biosynthesis of cinnamic acids is facilitated by cinnamate 4-hydroxylase (C4H), which catalyzes the conversion of cinnamate to *p*-hydroxycinnamate. Subsequently, 4-coumarate:CoA ligase (4CL) converts *p*-coumarates into their corresponding CoA esters.^20,21^

**Figure 2:**
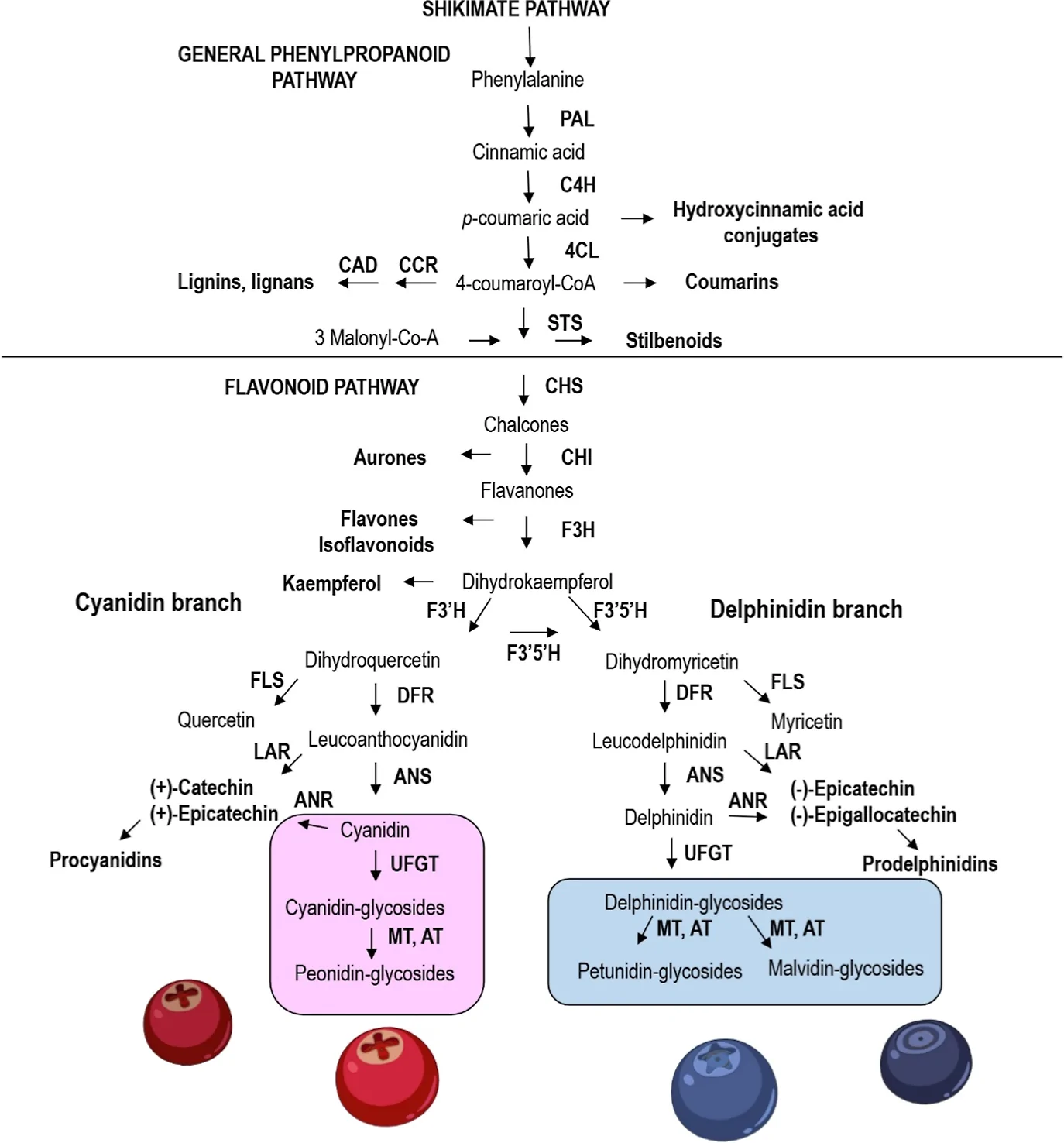
A schematic presentation of the phenylpropanoid and flavonoid pathway with emphasis on phenolic compounds present in *Vaccinium* berries. Red-colored *Vaccinium* berries contain primarily cyanidin branch anthocyanins whereas both cyanidin and delphinidin branch anthocyanins are present in blue-colored berries. Enzyme abbreviations: PAL, phenylalanine ammonia-lyase; C4H, cinnamate 4-hydroxylase; 4CL, 4-coumaroyl:CoA ligase; CAD, cinnamoyl-alcohol dehydrogenase; CCR, cinnamoyl- CoA reductase; STS, stilbene synthase; CHS, chalcone synthase; CHI, chalcone isomerase; F3H, flavanone 3-hydroxylase; F3′H, flavonoid 3′ hydroxylase; F3′5′H, flavonoid 3′5′ hydroxylase; FLS, flavonol synthase; DFR, dihydroflavonol 4-reductase; LAR, leucoanthocyanidin reductase; ANR, anthocyanidin reductase; ANS, anthocyanidin synthase; UFGT, UDP glucose-flavonoid 3-*O*-glucosyl transferase; MT, methyltransferase; AT, acyltransferase. Created in BioRender. Jaakola, L. (2026) https://BioRender.com/ap7fdk8.

The specific flavonoid pathway begins with the condensation of one molecule of 4-coumaroyl-CoA and three molecules of malonyl-CoA, resulting in the formation of naringenin chalcone (Figure 2).^21^ This reaction is catalyzed by chalcone synthase (CHS). Chalcone is then isomerized into (2S)-flavanone by chalcone flavanone isomerase (CHI). From these central intermediates, the pathway branches into various side routes, producing diverse classes of flavonoids. Flavanone 3-hydroxylase (F3H) catalyzes the stereospecific 3β-hydroxylation of (2S)-flavanones to dihydrokaempferol, which serves as a precursor for flavonols, anthocyanins, flavan-3-ols, and proanthocyanidins. The hydroxylation of the B-ring of dihydrokaempferol is carried out by flavonoid 3′-hydroxylase (F3′H) and flavonoid 3′,5′-hydroxylase (F3′5′H), producing dihydroquercetin or dihydromyricetin, which direct the biosynthesis toward the cyanidin or delphinidin branches, respectively. Flavonol synthase (FLS) catalyzes the dehydrogenation of dihydroflavonols into their corresponding flavonols. Competing with FLS, dihydroflavonol 4-reductase (DFR) facilitates the conversion of dihydroflavonols into leucoanthocyanins, which are subsequently transformed into anthocyanidins by anthocyanidin synthase (ANS). Glycosylation of anthocyanidins by UDP-glucose:flavonoid-*O*-glucosyltransferases (UFGT) results in the formation of anthocyanins, while methyltransferases (MT) are responsible for producing O-methylated anthocyanins such as peonidin, petunidin, and malvidin.^21,22^ Acylation of anthocyanins is carried out by acyltransferases (ATs), members of which have recently been characterized in blueberry.^23,24^

The monomeric units of proanthocyanidins, flavan-3-ols, are synthesized through two pathways: leucocyanidins are converted into (+)-catechins by leucoanthocyanidin reductase (LAR), while anthocyanidins are converted into (−)-epicatechins by anthocyanidin reductase (ANR) (Figure 2).^17^ Once synthesized, flavonoids are transported to vacuoles via vesicle trafficking, membrane transporters, and glutathione-S-transferase.^25^ The polymerization or oligomerization of proanthocyanidins occurs within the vacuoles. It has been unclear whether this process is enzymatic or nonenzymatic, however, recent studies support a nonenzymatic mechanism in which flavan-3-ol monomers serve as starter units for sequential trapping of extension units via nucleophilic attack. In this current PA polymerization model, LAR is a key enzyme in regulating the ratio of starter and extension units.^26^ Activity of both LAR and ANR has been reported in *Vaccinium* species corresponding to the chemical profiling of flavan-3-ols in these berries.^17,27^ In bilberry, one *ANR* gene and three homologues for LAR were identified from the sequence databases, all of which showed different expression profiles during berry ripening and in leaf, stem, and rhizome tissues, indicating diverse roles in PA biosynthesis.^17^

## 4 Transcriptional Regulation

In transcriptional level, biosynthesis of flavonoids is regulated via coordinated control of the structural enzymes in the pathway by the MYB-bHLH-WD40 (MBW) complex that includes interactions with DNA-binding R2R3MYB transcription factors, MYC-like basic helix loop helix (bHLH), and WD40-repeat proteins (Figure 3).^22,28^ The R2R3MYB is considered as the main component in the MBW complex specifying the target gene activation, even though bHLH partner can also have an role in the activation.^28^ The understanding of the transcriptional regulation of flavonoid biosynthesis has primarily been based on the studies performed in model species such as *Arabidopsis thaliana*, *Petunia hybrida*, *Antirrhinum majus* and red fruits of the Rosaceae family, which exhibit simpler anthocyanin profile compared with blue-colored berries. Studies on red grapes (*Vitis* spp.) and blue-colored *Vaccinium* berries have, however, advanced our understanding of the transcriptional regulation of the more complicated anthocyanin profiles including the delphinidin branch of the flavonoid pathway, with a particular focus on the role of MYB family members.^29–32^

Research focused on blue-colored *Vaccinium* berries has demonstrated that MYBA, MYBPA1, MYBPA2 subgroups, and MYB suppressors collaboratively regulate anthocyanin biosynthesis.^27,31–35^ In bilberry, 18 R2R3MYBs associated with flavonoid biosynthesis regulation have been identified.^34^ Among them, the MYBPA1-type transcription factor VmMYBPA1.1 was found to specifically regulate the delphinidin pathway by influencing the expression of the branching point gene *VmF3′5′H* and other genes involved in delphinidin biosynthesis, resulting in bluish anthocyanin pigments.^34^ Further studies revealed that in the early stage of fruit development, MYBPA2.3 activates MYBPA1.1 to promote PA biosynthesis, and at the onset of ripening, MYBA1 activates the MYBPA1.1 promoter to drive anthocyanin biosynthesis in both blueberry (*V. corymbosum*) and bilberry.^35^ Earlier, MYBPA1 transcription factors were generally associated with PA biosynthesis, even if increase in expression at the onset of ripening and anthocyanin production has been detected in blue-colored berries, such as Chinese bayberry (*Myrica rubra*) and grapevine (*Vitis* spp.) in addition to *Vaccinium* berries.^31,33,36–39^ The absence of these transcription factors in blueberry flesh explains its white coloration, in contrast to bilberry, which accumulates anthocyanins in both skin and flesh.^27^ In bilberry, altogether 8 PA type MYB transcription factor sequences were identified, representing MYBPA1, MYBPA2, and MYB3 subgroups.^34^ Besides the active role of MYBPA1.1 and MYBPA2.2 connected to anthocyanin biosynthesis in ripening berry, the precise function of all others has not yet been fully characterized. Expression of these regulators is higher in the early development of berries and in leaf, stem, and root tissues. It seems that VmMYBPA1.2 has the conventional role of MYBPA1 transcription factors in regulation of proanthocyanidin biosynthesis as overexpression of this gene in *Nicotiana benthamiana* leaves strongly upregulated *NbANR* gene.^34^

Concerning other MYB regulators, also MYB5 members contributing to precursors of the flavonoid pathway have been identified in *Vaccinium* berries in addition to MYBC group repressors.^33,34^ It has been shown earlier that MYBC-type repressors can provide feedback inhibition in the MBW complexes associated with anthocyanin biosynthesis for fine-tuning the pigment production.^40^ In *Vaccinium* species, precise function of the MYBC group repressors has not yet been studied in detail.

Additionally, the recent characterization of bilberry bHLH transcription factors provides new insights into the interaction between R2R3-MYBs and bHLH partners within the MBW complex.^41^ The results suggest that TT8-type VmbHLH2 is the main family member involved in anthocyanin biosynthesis during the bilberry ripening.

Concerning upstream regulators, a number of transcription factors from MADS-box, NAC, SPL, and WRKY families have been shown to regulate fruit development and ripening. Of these, SQUAMOSA MADS-box gene TDR4/FUL1 was shown to have a role in accumulation of anthocyanin during bilberry ripening.^30^ Moreover, several ripening regulator candidates have been identified from the recent *Vaccinium* transcriptomes and genomes.^42–48^ Recently, transient injection of a blueberry ethylene response factor (ERF) transcription factor, a member of APETALA2/ETHYLENE RESPONSIVE FACTOR (AP2/ERF) family, on blueberry leaves and fruits increased anthocyanin biosynthesis.^49^ Similar response was found in transgenic adventitious shoots of the same gene.^49^

Epigenetic regulation including DNA methylation and histone modifications has been shown to have a role in anthocyanin accumulation in fruits. For instance, in apple (*Malus domestica*) and pear (*Pyrus* spp.), DNA methylation in the promoter region of the R2R3-MYB transcription factor of the MBW complex was found to negatively regulate anthocyanin biosynthesis.^73^ In *Vaccinium* berries, DNA methylation of the promoter regions of structural genes *VcCHS* and *VcANS* was shown to inhibit anthocyanin biosynthesis in blueberry (*V. corymbosum*).^74^

## 5 Hormonal Regulation

Plant hormones regulate fruit development and ripening, coordinating physiological and biochemical transitions between different stages. Auxins and gibberellins are involved in the regulation of fruit set and early development, whereas ethylene and abscisic acid (ABA) are the key-regulations of the ripening process. Based on the respiration burst rate and increase in ethylene production or the ABA peak at the onset of ripening, fruits are classified into climacteric or nonclimacteric fruits.^50^ *Vaccinium* berries are generally classified as nonclimacteric fruits, however, evidence on both ethylene and ABA accumulation has been detected at the onset of ripening, which indicates that both of these plant hormones have a role in the ripening process.^51^ Watanabe et al. pointed out the controversy over the classification of blueberries (*Vaccinium* spp.) as climacteric or nonclimacteric fruit because some of the earlier studies have detected increased respiration rate and ethylene production during the maturation period while some other studies did not observe ethylene evolution but only increase in ABA concentration in ripening blueberries.^39,52,53^ Especially ABA has been found to be connected and to induce ripening-related anthocyanin accumulation in bilberries and blueberries.^39,54–56^ It has also been shown that red light can stimulate anthocyanin biosynthesis regulated by MBW complex via induction of the ABA biosynthesis.^10,57^ It seems that the burst of ethylene at the onset of berry ripening increases ABA biosynthesis, leading to increase in anthocyanin biosynthesis.^56^ Indeed, in rabbiteye blueberry (*V. ashei*) fruit, a treatment with ethephon, an ethylene-releasing compound, induced increase in the ABA levels and related anthocyanin biosynthesis.^58^

Sugar-hormone cross talk has been shown to have a role in regulation of anthocyanin biosynthesis, and sugar accumulation has been found to stimulate anthocyanin biosynthesis in different models, such as in *Arabidopsis* leaves and in detached grape (*Vitis* spp.) berries or in vitro cultures.^75^ In strawberry (*Fragaria x ananassa*), exogenous sucrose treatments in postharvest fruit increased pelargonidin-derivative anthocyanins and related gene expression.^76^ In *Vaccinium* berries, accumulation of sugars fructose, glucose, and sucrose occurs simultaneously with anthocyanin accumulation during berry ripening.^6,77–79^ However, Karppinen et al. tested exogenous sucrose and glucose treatments of green bilberry fruits in the same study with exogenous treatment of berries with two different ABA concentrations.^55^ ABA effectively induced anthocyanin accumulation in 4 days, whereas no statistical differences were detected in sucrose- or glucose-treated berries compared to controls.

## 6 Environmental Regulation

Environmental factors have a considerable role in the regulation of secondary metabolism in general and specifically of phenolic compounds. Light and temperature conditions in addition to nutritional status, water balance, diseases, and pest attacks have all been shown to affect the biosynthesis of phenolic compounds.^59^ However, interaction of different stressors affects differentially different species and cultivars because of which controlled studies can bring valuable information. Especially, the effect of light and temperature on the metabolism of phenolic compounds has been widely studied in both wild and cultivated *Vaccinium* species.

Light intensity and quality, in addition to photoperiod have all been shown to affect profile of phenolic compounds in *Vaccinium* species (Figure 3). In many studies, higher light intensity has been shown to increase the content of phenolic compounds.^60–62^ However, strong interaction has been detected between temperature, and light intensity as bilberries adapted to high altitudes produced lower levels of anthocyanins in field conditions at low altitude in northern Italy, compared to the plants under blue or black selective nets, which offered slightly lower temperature.^63^ In separate studies light quality has been shown to markedly affect anthocyanin biosynthesis.^57,64,65^ Samkumar et al. showed that both red and blue light significantly increase anthocyanin biosynthesis in ripening bilberries, however, the response was different if berries were attached or detached on the mother plant during the light treatments.^57,66^ In the same study, red light also increased levels of most flavonols in addition to catechin and gallocatechin levels but not as much as anthocyanins.^66^ Transcriptome analyses of the samples revealed that the red light signaling induced ABA-regulated anthocyanin biosynthesis.^57^ Longer day length has also been shown to increase accumulation of anthocyanins in ripening bilberry.^67^ Based on these studies, it seems that anthocyanins, especially delphinidin branch anthocyanins, react more in higher light intensity or increase in red and blue light, compared to other flavonoids or phenolic compounds in *Vaccinium* berries.

*Vaccinium* species and cultivars have adapted to different temperature regimes at different growth regions. Southern highbush blueberries can be grown in warmer temperatures, such as in Mediterranean climate region, whereas half-high and lowbush blueberries, as well as cranberries, lingonberries, and bilberries prefer cooler growth temperatures. Both bilberries and lingonberries have been shown to produce higher levels of phenolic compounds in lower temperatures under controlled conditions.^6,67^ Moreover, many studies have indicated higher anthocyanin levels in bilberries, bog bilberries, and lingonberries growing in higher latitudes characterized with cooler temperature and longer day length, compared to the southern counterparts.^6,68–70^ Same phenomenon has been detected toward higher altitudes.^71,72^ Considering the ongoing global warming, it seems that in the future, the warmer climate during the growth season may change the composition and decrease the content of the key phenolic compounds especially of the wild *Vaccinium* berries adapted to northern climates. Moreover, in the southern areas, the wild *Vaccinium* species may be restricted even in higher altitudes, reducing the present distribution areas.

In conclusion, a great deal of information has become available on factors affecting the accumulation of phenolic compounds in *Vaccinium* species. Especially understanding the regulation of the biosynthesis of phenolic compounds in *Vaccinium* berries has advanced markedly in recent years. Candidates of the MBW complex have been identified and characterized in different species, and functional studies have increased understanding of the transcriptional regulation, especially of blue-colored *Vaccinium* berries. Many recent *Vaccinium* genomes and transcriptomes are available for blueberries, cranberries, bilberries, and lingonberries, which are detailedly described by Edger et al.^80^ These databases offer further possibilities to deepen understanding of the complex regulation and signaling networks regarding hormonal and environmental regulation. More detailed studies are needed to clarify the interaction of ethylene and ABA during berry ripening and the effect of the interaction on berry quality in different types of *Vaccinium* berries. Along with a deeper understanding of the environmental and hormonal regulation, it is possible to develop new tools for the selection of suitable cultivars for different climate regions. Improving the health properties and quality of *Vaccinium* berries can be targeted via the breeding programs but also by paying attention to growth conditions, for instance, by choosing an optimal growth location, optimizing light conditions in tunnel systems, or reflecting shields or shading nets.

**Figure 3:**
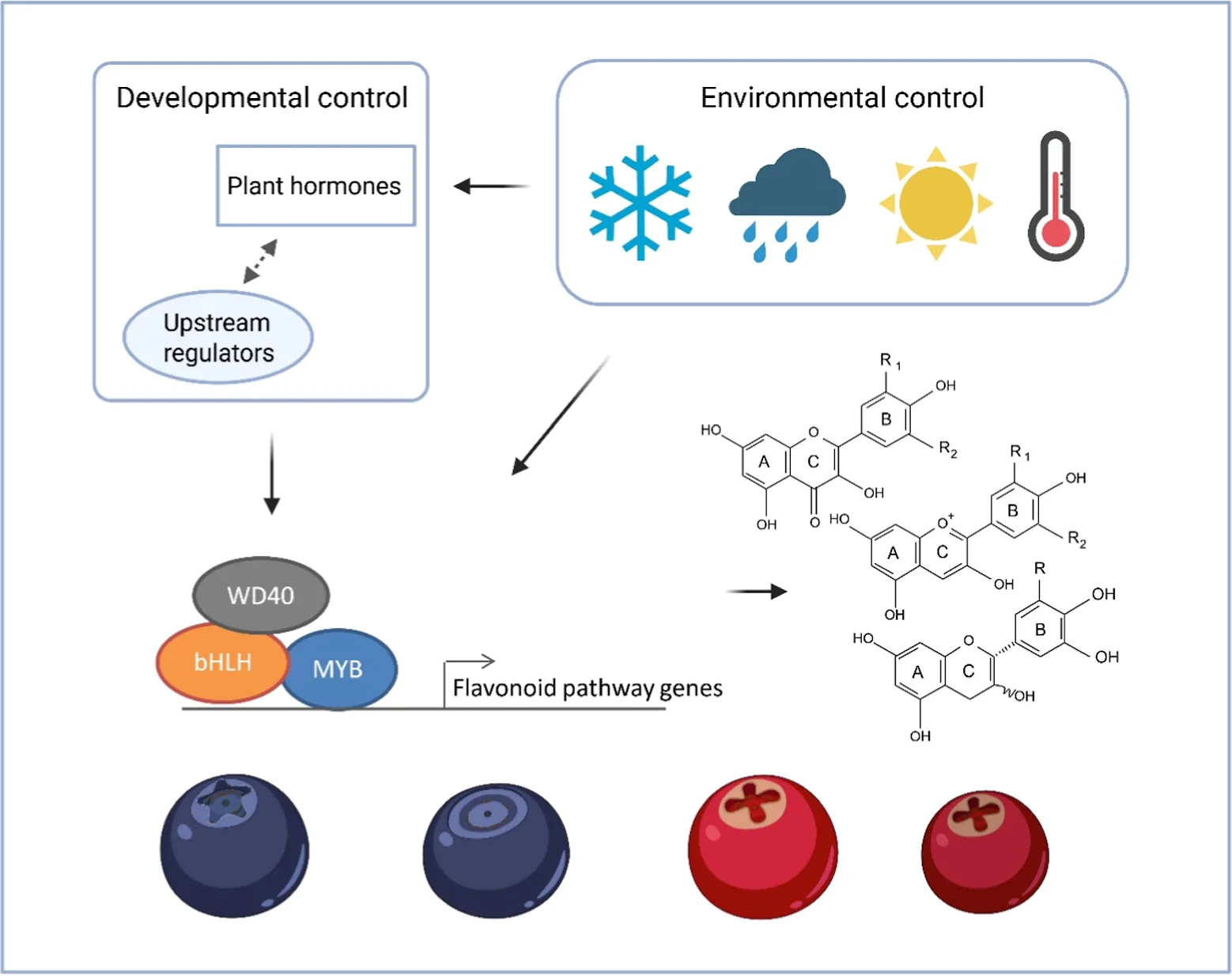
A schematic presentation on the regulation of the biosynthesis of phenolic compounds in *Vaccinium* berries focusing on flavonoid group phenolics. Created in BioRender. Jaakola, L. (2026) https://BioRender.com/jb6vs9e.

## Abbreviations

AT: acetyltransferase
ABA: abscisic acid
ANR: anthocyanidin reductase
ANS: anthocyanidin synthase
bHLH: MYC-like basic helix loop helix
CHI: chalcone flavanone isomerase
CHS: chalcone synthase
4CL: 4-coumarate:CoA ligase
DFR: dihydroflavonol 4-reductase
F3H: flavanone 3-hydroxylase
F3′H: flavonoid 3′-hydroxylase
F3′5′H: flavonoid 3′,5′-hydroxylase
FLS: flavonol synthase
FUL1: FRUITFULL 1
HCA: hydroxycinnamic acid
LAR: leucoanthocyanidin reductase
MYB: DNA-binding R2R3MYB transcription factor
MBW: MYB-bHLH-WD40
MT: methyltransferase
PA: proanthocyanidin
SPL: SQUAMOSA promoter-binding protein like
STS: stilbene synthase
TDR4: tomato FRUITFULL homologue 1
UFGT: UDP-glucose:flavonoid-*O*-glucosyltransferase
WD40: WD40-repeat proteins
